# Reliability of the Spinal Instability Neoplastic Score (SINS) among radiation oncologists: an assessment of instability secondary to spinal metastases

**DOI:** 10.1186/1748-717X-9-69

**Published:** 2014-03-04

**Authors:** Charles G Fisher, Rowan Schouten, Anne L Versteeg, Stefano Boriani, Peter Pal Varga, Laurence D Rhines, Norio Kawahara, Daryl Fourney, Lorna Weir, Jeremy J Reynolds, Arjun Sahgal, Michael G Fehlings, Ziya L Gokaslan

**Affiliations:** 1 Division of Spine, Department of Orthopaedics, University of British Columbia and Vancouver Coastal Health, Vancouver, BC, Canada; 2 Department of Orthopaedic Surgery and Musculoskeletal Medicine, Christchurch Hospital, Christchurch, New Zealand; 3 Department of Orthopaedics, University Medical Center Utrecht, Utrecht, Netherlands; 4 Department of Degenerative and Oncological Spine Surgery, Rizzoli Institute, Bologna, Italy; 5 National Center for Spinal Disorders and Buda Health Center, Budapest, Hungary; 6 Department of Neurosurgery, MD Anderson Cancer Center, The University of Texas, Houston, TX, USA; 7 Department of Orthopaedic Surgery, Kanazawa Medical University, Kahoku-gun, Japan; 8 Division of Neurosurgery, University of Saskatchewan, Royal University Hospital, Saskatoon, SK, Canada; 9 BC Cancer Agency, Vancouver Centre and Department of Medicine, University of British Columbia, Vancouver, BC, Canada; 10 Spinal Unit, Oxford University Hospital NHS Trust, Oxford, UK; 11 Department of Radiation Oncology, Princess Margaret Cancer Centre, University of Toronto, Toronto, ON, Canada; 12 Department of Radiation Oncology, Sunnybrook Health Sciences Center, University of Toronto, Toronto, ON, Canada; 13 Division of Neurosurgery, Department of Surgery, University of Toronto, Toronto, ON, Canada; 14 Krembil Neuroscience Centre, Toronto Western Hospital, Toronto, ON, Canada; 15 Department of Neurosurgery, Johns Hopkins University, School of Medicine, Baltimore, MD, USA; 16 Blusson Spinal Cord Centre, 6th Floor – 818 W10th Avenue, Vancouver V5Z 1M9 BC, Canada

**Keywords:** Neoplasm metastasis, Spine, Instability, Radiation oncology, Reliability and validity

## Abstract

**Background:**

The Spinal Instability Neoplastic Score (SINS) categorizes tumor related spinal instability. It has the potential to streamline the referral of patients with established or potential spinal instability to a spine surgeon. This study aims to define the inter- and intra-observer reliability and validity of SINS among radiation oncologists.

**Methods:**

Thirty-three radiation oncologists, across ten international sites, rated 30 neoplastic spinal disease cases. For each case, the total SINS (0-18 points), three clinical categories (stable: 0-6 points, potentially unstable: 7-12 points, and unstable: 13-18 points), and a binary scale (‘stable’: 0-6 points and ‘current or possible instability’; surgical consultation recommended: 7-18 points) were recorded. Evaluation was repeated 6-8 weeks later. Inter-observer agreement and intra-observer reproducibility were calculated by means of the kappa statistic and translated into levels of agreement (slight, fair, moderate, substantial, and excellent). Validity was determined by comparing the ratings against a spinal surgeon’s consensus standard.

**Results:**

Radiation oncologists demonstrated substantial (κ = 0.76) inter-observer and excellent (κ = 0.80) intra-observer reliability when using the SINS binary scale (‘stable’ versus ‘current or possible instability’). Validity of the binary scale was also excellent (κ = 0.85) compared with the gold standard. None of the unstable cases was rated as stable by the radiation oncologists ensuring all were appropriately recommended for surgical consultation.

**Conclusions:**

Among radiation oncologists SINS is a highly reliable, reproducible, and valid assessment tool to address a key question in tumor related spinal disease: Is the spine ‘stable’ or is there ‘current or possible instability’ that warrants surgical assessment?

## Background

Spinal column metastases are present in up to 70% of cancer patients and as life expectancy improves with advances in oncology treatment, rates are expected to increase [1-3]. The modalities available to treat patients with metastatic spine disease continue to evolve and are offered by a range of healthcare professionals including radiation and medical oncologists, radiologists, pain specialists, and surgeons. The aim of these treatments is to optimize a patient’s quality of life by providing effective pain relief and preserving or restoring neurological function.

Consultation with spine surgeons is typically initiated when neurological decompression or stabilization of impending or existing spinal instability is considered necessary. The Spinal Oncology Study Group (SOSG), consisting of 30 international spine oncology surgeons, recently defined spinal instability as “… loss of spinal integrity as a result of a neoplastic process that is associated with movement-related pain, symptomatic or progressive deformity, and/or neural compromise under physiological loads” [4]. Although vitally important, the assessment of tumor related spinal instability for the spine surgeon is challenging despite it being part of their training and practice. Being outside the surgical realm, instability recognition for the oncologist and other non-surgical members of the multidisciplinary care team is probably even more demanding; possibly resulting in under-recognition and under-referral of patients who may benefit from surgical intervention [5,6].

**Figure 1:**
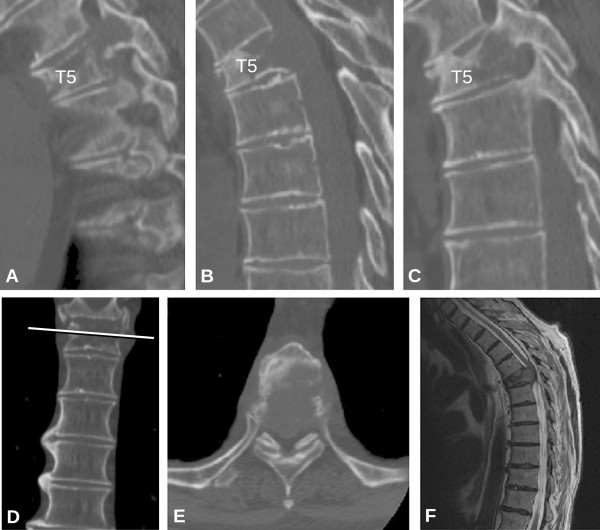
**A 67-year-old man with known metastatic small cell lung cancer presents with interscapular back pain that is exacerbated by movement and relieved with rest.** Computed Tomography (CT) (**A**: left parasagittal, **B**: midline parasagittal, **C**: right parasagittal, **D**: coronal, and **E**: axial) and Magnectic Resonance (MR) images (**F**: sagittal, T2-weighted) outline the key details of this T5 lesion. Total SINS score = Semirigid (T5) spine, 1 point; ‘Mechanical’ pain (yes), 3 points; Lytic lesion, 2 points; De novo kyphotic deformity without subluxation/translation, 2 points; >50% vertebral body collapse, 3 points; and bilateral posterolateral spinal element involvement, 3 points. Total SINS score = 1 + 3 + 2 + 2 + 3 + 3 = 14 (unstable, surgical consultation is recommended).

Recently, the SOSG developed the Spinal Instability Neoplastic Score (SINS), a standardized framework to help physicians assess and categorize spinal instability [7]. The purpose of SINS is to provide a tool to facilitate referrals for assessment and possible treatment of instability, a condition that can lead to catastrophic neurological, deformity and painful situations. The SINS evaluates spinal stability by adding together six radiographic and clinical components, with a score ranging from 0 to 18 (Table 1). A clinical example is shown in Figure 1. The total score is divided in three categories of stability: stable (0-6 points), potentially unstable (7-12 points), and unstable (13-18 points). In addition, the SINS score can also be analyzed as a binary indicator of surgical referral status: ‘stable’ (0-6 points) or ‘current or possible instability’ (7-18 points). Surgical consultation is recommended for those patients with a score of ≥7 (Table 2).

**Table 1:** **The SINS classification according to Fisher et al. [**[4]**]**

	**Score**
**Location**	
Junctional (occiput-C2, C7-T2, T11-L1, L5-S1)	3
Mobile spine (C3-C6, L2-L4)	2
Semirigid (T3-T10)	1
Rigid (S2-S5)	0
Pain^*^	
Yes	3
Occasional pain but not mechanical	1
Pain-free lesion	0
**Bone lesion**	
Lytic	2
Mixed (lytic/blastic)	1
Blastic	0
**Radiographic spinal alignment**	
Subluxation/translation present	4
De novo deformity (kyphosis/scoliosis)	2
Normal alignment	0
**Vertebral body collapse**	
> 50% collapse	3
< 50% collapse	2
No collapse with > 50% body involved	1
None of the above	0
**Posterolateral involvement of spinal elements**^**†**^	
Bilateral	3
Unilateral	1
None of the above	0

*Pain improvement with recumbency and/or pain with movement/loading of spine.

^†^Facet, pedicle, or costovertebral joint fracture or replacement with tumor.

**Table 2:** SINS scores organized as a total score, three-clinical categories, and binary scale with their corresponding levels of stability where surgical consultation is recommended for a total score ≥ 7

**Total Score (0-18 SINS)**	**1**	**2**	**3**	**4**	**5**	**6**		**7**	**8**	**9**	**10**	**11**	**12**		**13**	**14**	**15**	**16**	**17**	**18**
**Three Clinical Categories (3-point)**	Stability							Potentially Unstable							Unstable					
**Binary Scale (2-point)**	Stability							Current or potential instability; Surgical consultation recommended												

The SINS performance was previously tested among members of the SOSG. This study revealed a sensitivity and specificity for potentially unstable or unstable lesions of 95.7% and 79.5%, respectively. It demonstrated near-perfect inter- and intra-observer reliability for differentiating the three clinical categories of stability [8]. Patients with spinal metastases receive the majority of their care from oncologists who often find evaluating stability difficult. Therefore, before the widespread use of SINS can be recommended and studied prospectively, its reliability and validity has to be established among oncologists. The purpose of this study is to assess the reliability and validity of the SINS score among radiation oncologists. We envision that the SINS can be used in the future to assist in directing appropriate evaluation and referral of these complex patients to spine surgeons when necessary.

## Methods

### Patient cases

A radiation oncologist and spinal surgeon not related to or participating in the study compiled 30 de-identified cases of metastatic spine disease. The cases were comprised of 13 females and 17 males, with ages ranging from 40-79 and were reviewed at ten international sites. Twelve primary pathologies were represented with lung (*n* = 6), prostate (*n* = 6), breast (*n* = 5), and myeloma (*n* = 4) being most popular. Lesions were spread across the cervical (C0-C7) spine (*n* = 6), thoracic (T1-T10) spine (*n* = 11), thoracolumbar (T11-L2) junction (*n* = 8), and low lumbar (L3-L5) regions (*n* = 5). Information provided included demographic data, histopathological diagnosis, and relevant clinical information including a pain description that emphasized the relationship of the pain to activity and rest; the purpose being to differentiate oncologic from mechanical or movement related pain. Plain radiographs, Computed Tomography (CT) images, and Magnetic Resonance (MR) images followed. CT scans were provided for each assessment.

To determine a validity reference or surgical consensus standard, 11 fellowship trained oncology spine surgeons from both neurosurgical and orthopedic backgrounds, all experienced with the SINS classification, rated the cases once. None of the raters had been involved in the care of any of the cases. The consensus standard score was the value that occurred most frequently. In cases where ties occurred, the two most frequent scores were averaged to calculate the final value.

After undergoing an instructional session and case examples on the application of the SINS, 33 radiation oncologists from North America, Europe, and Asia independently scored the SINS on the same 30 cases. Six to eight weeks later, after the order of cases was changed to minimize recall bias, the cases were scored again. The radiation oncologists received a stipend for scoring the cases. None of the cases in the instructional session were used in the actual study cases.

For each case, the SINS score was rated and analyzed as a total (0-18) point score, as three clinical categories (0-6 points indicating a stable spine, 7-12 points indicating potentially unstable spine, and 13-18 points indicating an unstable spine), and as a binary scale (‘stable’ (0-6 points) or ‘current or possible instability’ (7-18 points)) (Table 2). This study received ethics committee approval.

### Statistical analysis

Inter-observer agreement was calculated for both the radiation oncologists and the surgeons based on the first review using the Fleiss’ kappa for multiple raters [9]. Confidence intervals for the kappa estimates were calculated by means of bootstrapping, simulating 1,000 kappa estimates, and using the 2.5 and 97.5 percentiles for the confidence intervals. Intra-observer reproducibility was assessed for the radiation oncologists using the average Cohen’s kappa [10]. The kappa coefficients (κ) were calculated for the three clinical categories (3-point scale), and the binary scale (2-point scale) and interpreted according to the widely accepted Landis and Koch [11] grading system (Table 3). The intraclass correlation coefficient (ICC) was used to measure both the inter- and intra-observer agreement for the total point score. As a measure of validity, the average kappa for agreement between the radiation oncologists’ rating and the surgeon’s consensus standard was used. The magnitude of agreement guidelines of the kappa, ranging from “no agreement” to “almost perfect agreement”, was also used (Table 3). All statistical analyses were performed with Stata 12.0 for Windows (StataCorp LP, College Station, TX, USA).

**Table 3:** Levels of agreement for κ statistic levels

**κ Value**	**Level of agreement**
0.00-0.20	Slight
0.21-0.40	Fair
0.41-0.60	Moderate
0.61-0.80	Substantial
> 0.80	Excellent

NOTE. Data adapted from Landis and Koch [11].

### Role of the funding source

AOSpine International provided resources necessary to conduct this study including stipends to the radiation oncologists who reviewed the cases. They facilitated strategic project meetings with the co-authors and assisted in the study management process. The authors were not compensated.

## Results

### Inter-observer agreement

Among surgeons, substantial (κ = 0.65) agreement for the three clinical categories and excellent (κ = 0.83) agreement for the binary scale was achieved. The agreement among the radiation oncologists for the three clinical categories and binary scale was moderate (κ = 0.54) and substantial (κ = 0.76), respectively (Table 4). Inter-observer agreement of the total point score was ICC = 0.85 among the surgeons and ICC = 0.80 among radiation oncologists. Among radiation oncologists, analysis of the separate SINS components showed excellent level of agreement for location (κ = 0.94) and pain (κ = 0.88) and moderate agreement for bone lesion quality (κ = 0.55), spinal alignment (κ = 0.42), vertebral body collapse (κ = 0.57), and posterolateral involvement of the spinal elements (κ = 0.43).

**Table 4:** Reliability analysis of the SINS among radiation oncologists and spine surgeons

**Profession**	**Inter-observer agreement**		**Intra-observer reproducibility**	
	**Three-point scale (95% CI)**	**Binary scale (95% CI)**	**Three-point scale (95% CI)**	**Binary scale (95% CI)**
Radiation oncologists	0.54 (0.40-0.64)	0.76 (0.56-0.88)	0.65 (0.60-0.71)	0.80 (0.74-0.86)
Spine surgeons (Gold standard)	0.65 (0.50-0.75)	0.83 (0.65-0.93)	n.a.	n.a.

### Intra-observer reproducibility

Among radiation oncologists, intra-observer reproducibility was substantial (κ = 0.65) for the three clinical categories and excellent (κ = 0.80) for the binary scale (Table 4). Intra-observer reproducibility of the total point score among radiation oncologists was ICC = 0.88. Analysis of the separate SINS components showed excellent reproducibility for location (κ = 0.96) and pain (κ = 0.91), substantial consistency for bone lesion quality (κ = 0.68), spinal alignment (κ = 0.63), and vertebral body collapse (κ = 0.63), and moderate agreement for posterolateral involvement of the spinal elements (κ = 0.58).

### Validity

The level of agreement (average Cohen’s kappa) between the spine surgeon’s consensus standard and the radiation oncologists for the three clinical categories was 0.61 and 0.85 for the binary scale. Table 5 outlines the three clinical categories (0-6 stable, 7-12 potentially unstable, and 13-18 unstable) rated by the radiation oncologists cross-tabulated with the spine surgeons opinion, the gold standard. None of the unstable cases and only 18 of 561 (3%) of the potentially unstable cases, as determined by spine surgeon’s consensus standard, was rated as stable by the radiation oncologists. These potentially unstable and unstable cases were rated a score (≥7) for which surgical consultation is may be prudent. Fourteen percent of cases rated stable by the surgeons were considered potentially unstable by the oncologists.

**Table 5:** Cross tabulation of scores determined by the gold standard and categorization by radiation oncologists

	**Gold standard**			**Total**
**Three-point Scale SINS by Radiation Oncologists**	**Stable**	**Potentially unstable**	**Unstable**	
**Stable**	170	18	0	188
**Potentially Unstable**	28	409	56	493
**Unstable**	0	134	175	309
**Total**	198	561	231	990

Gold Standard: score occurred most frequently among spine surgeons. Radiation oncologist first assessment (N = 30).

## Discussion

Metastatic spine disease requires a multidisciplinary approach in order to provide optimal care; therefore, it is important to establish a clear framework and terminology when describing important parameters such as spinal stability to avoid variability in interpretation, referral patterns and consequently patient management. SINS is a highly reliable, reproducible and provisionally valid assessment tool for radiation oncologists attempting to answer a key question in tumor related spinal disease: Is the spine ‘stable’ or is there ‘current or potential instability’ that warrants surgical assessment? Based on the current study, we propose that the SINS should become part of the routine clinical assessment of patients with symptomatic spinal metastases. Although SINS has not undergone prospective clinical evaluation, it has been derived through evidence based medicine methodology and is the closest thing to a gold standard we have. In development of a classification or tool such as SINS, it must be assessed for face, content and criterion validity, along with reliability. With these established SINS can undergo prospective validation and only then, if successful, will it be a true gold standard.

The agreement for each individual component of the SINS score varied between ‘moderate’ and ‘excellent’. In part, this may be attributable to the study design with limited information provided for each case compared to the true clinical situation. Trying to accurately mimic real practice is one of the inherent limitations of doing reliability studies and could serve to increase or decrease reliability. For example, more history would be obtainable from ‘real’ patients with respect to movement-related pain that may strengthen agreement. Conversely, history taking varies and patients are sometimes vague and do not have a distinct pain character and this could increase variability. The authors are hopeful that SINS will increase awareness of movement related pain and lead to accurate capture of this important variable. In the study there was excellent reproducibility on the pain component (κ = 0.91). Only moderate agreement was recorded for bone lesion quality, spinal alignment, vertebral body collapse, and posterolateral involvement of the spinal elements. Similar findings were noted when the reliability of the SINS was tested among members of the SOSG [8]. We acknowledge that these components may have a greater degree of subjective interpretation but it may reflect the availability of appropriate imaging or the inability to scroll through reformatted images in the study setting. On the other hand, having representative images selected for the study could improve reliability. Similarly in real practice, scrolling through reams of images to obtain representative ones could lead to increased variability. Imperfect agreement on location may reflect a discrepancy in application of SINS or illustrate limitations in anatomical interpretation of the static images provided. The only way these limitations will be truly assessed is during prospective evaluation in a clinical setting. While agreement between surgeons and radiation oncologists was favorable for the total (0-18) point score, this measure is far less clinically relevant than the binary scale. Finally, the radiation oncologists’ lack of familiarity with some of the terms in SINS may also impact reliability.

Many scoring systems aiming to select patients that may benefit from spinal surgery for metastatic disease exist. Almost all attempt to guide decision-making by predicting survival prognosis [12-17]. In addition to prognosis, other key factors require consideration, including spinal stability. Spinal metastases can cause spinal instability, which may present as severe pain, progressive deformity and/or neurological compromise, all potential indications for surgical intervention. Prior to the development of the SINS classification the assessment of spinal integrity lacked standardization and proved difficult especially for non-surgical members of the multidisciplinary care team. The goal of the SINS classification is to provide a framework that helps all healthcare professionals identify patients with impending or existing spinal instability who may benefit from surgical intervention. A SINS score of 7 or greater should prompt consultation with a spine surgeon, who can highlight the available surgical options during collaborative discussions on a patient’s optimal treatment. A potential limitation of this however is too many unnecessary referrals, which is likely not economically responsible. Fourteen percent of cases rated stable by the surgeons were considered potentially unstable by the radiation oncologists. This must be assessed with SINS’ prospective evaluation in a multicenter clinical setting.

Reliability of the SINS has already been evaluated among members of the SOSG [8]. Before widespread clinical implementation the reliability and validity of the SINS should be investigated across healthcare specialties. A similar assessment of the SINS classification with radiologists has also been initiated. SINS may also prove valuable beyond the clinical setting as a research tool to better classify patients and as instability is likely a strong confounder for several outcomes in treatment evaluation.

Our study showed substantial inter-observer and excellent intra-observer reliability for the binary SINS scale (stable versus unstable/potentially unstable) among radiation oncologists. Thirty cases were reviewed twice by 33 radiation oncologists at ten international sites, with sufficient power (>94%) [18]. Generalizability was enhanced by a multicenter, international group of oncologists who reviewed cases that represented the established epidemiological profile of metastatic spine disease. SINS is designed to be reliable and valid throughout the mobile spine.

With appropriate diagnostic test parameter testing completed, steps will be taken to implement SINS integration into clinical practice so multicenter prospective evaluation can be carried out. With an evidence based medicine process to develop SINS, followed by reliability and validity studies involving the key specialists who will use it, adoption into clinical practice should be feasible. Furthermore, it would appear limited training is necessary based on the reliability and validity performance of SINS amongst radiation oncologists after a brief instruction and case example tutorial.

## Conclusions

The Spinal Instability Neoplastic Score (SINS) is a simple, reliable, and valid tool to determine metastatic spinal instability and facilitate appropriate surgical referral. This study has shown that among radiation oncologists, the SINS binary scale provides a reliable tool for rating tumor-related spinal instability.

## Abbreviations

CI: Confidence interval; CT: Computed tomography; ICC: Intraclass correlation coefficient; MR: Magnetic resonance; SINS: Spinal instability neoplastic score; SOSG: Spinal Oncology Study Group.

## Competing interests

This research was supported by AOSpine International through the Knowledge Forum Tumor. AOSpine is a clinical division of the AO Foundation—an independent medically-guided not for profit organization.

## Authors’ contributions

Author study contribution and manuscript preparation include the following: Conception and design: CF, SB, PV, LR, MF, ZG DF; Acquisition of data: CF, RS, SB, PV, LR, NK, DF, LW, JR, AS, MF, ZG; Analysis and interpretation: CF, RS, AV, SB, PV, LR, JR, ZG; Drafting the article: CF, RS, AV; Critically revising the article: all authors; Reviewed submitted version of manuscript: all authors. All authors read and approved the final manuscript.

## References

[B1] Falicov A Fisher CG Sparkes J Boyd MC Wing PC Dvorak MF Impact of surgical intervention on quality of life in patients with spinal metastases Spine 2006 31 24 2849 2856 10.1097/01.brs.0000245838.37817.40 17108840

[B2] Hayat MJ Howlader N Reichman ME Edwards BK Cancer statistics, trends, and multiple primary cancer analyses from the Surveillance, Epidemiology, and End Results (SEER) Program Oncologist 2007 12 1 20 37 10.1634/theoncologist.12-1-20 17227898

[B3] Harel R Angelov L Spine metastases: Current treatments and future directions Eur J Cancer 2010 46 15 2696 2707 10.1016/j.ejca.2010.04.025 20627705

[B4] Fisher CG DiPaola CP Ryken TC Bilsky MH Shaffrey CI Berven SH Harrop JS Fehlings MG Boriani S Chou D Schmidt MH Polly DW Biagini R Burch S Dekutoski MB Ganju A Gerszten PC Gokaslan ZL Groff MW Liebsch NJ Mendel E Okuno SH Patel S Rhines LD Rose PS Sciubba DM Sundaresan N Tomita K Varga PP Vialle LR A novel classification system for spinal instability in neoplastic disease: an evidence-based approach and expert consensus from the Spine Oncology Study Group Spine 2010 5 E1221 E1229 20562730 10.1097/BRS.0b013e3181e16ae2

[B5] Eastley N Newey M Ashford RU Skeletal metastases – The role of the orthopaedic and spinal surgeon Surg Oncol 2012 21 3 216 222 10.1016/j.suronc.2012.04.001 22554913

[B6] Galasko CS Norris HE Crank S Spinal instability secondary to metastatic cancer J Bone Joint Surg Am 2000 82 4 570 594 10761947 10.2106/00004623-200004000-00012

[B7] Weber MH Burch S Buckley J Schmidt MH Fehlings MG Vrionis FD Fisher CG Instability and impending instability of the thoracolumbar spine in patients with spinal metastases: a systematic review Int J Oncol 2011 38 1 5 12 21109920

[B8] Fourney DR Frangou EM Ryken TC Dipaola CP Shaffrey CI Berven SH Bilsky MH Harrop JS Fehlings MG Boriani S Chou D Schmidt MH Polly DW Biagini R Burch S Dekutoski MB Ganju A Gerszten PC Gokaslan ZL Groff MW Liebsch NJ Mendel E Okuno SH Patel S Rhines LD Rose PS Sciubba DM Sundaresan N Tomita K Varga PP Spinal instability neoplastic score: an analysis of reliability and validity from the spine oncology study group J Clin Oncol 2011 29 22 3072 3077 10.1200/JCO.2010.34.3897 21709187

[B9] Fleiss JL Measuring nominal scale agreement among many raters Psychol Bull 1971 76 378 381

[B10] Cohen J A coefficient of agreement for nominal scales Educ Psychol Meas 1960 20 37 46 10.1177/001316446002000104

[B11] Landis JR Koch GG The measurement of observer agreement for categorical data Biometrics 1977 33 159 174 10.2307/2529310 843571

[B12] Bauer HC Wedin R Survival after surgery for spinal and extremity metastases. Prognostication in 241 patients Acta Orthop Scand 1995 66 143 146 10.3109/17453679508995508 7740944

[B13] Sioutos PJ Arbit E Meshulam CF Galicich JH Spinal metastases from solid tumors. Analysis of factors affecting survival Cancer 1995 76 1453 1459 10.1002/1097-0142(19951015)76:8<1453::AID-CNCR2820760824>3.0.CO;2-T 8620423

[B14] Tokuhashi Y Matsuzaki H Oda H Oshima M Ryu J A revised scoring system for preoperative evaluation of metastatic spine tumor prognosis Spine 2005 30 2186 2191 10.1097/01.brs.0000180401.06919.a5 16205345

[B15] Tokuhashi Y Matsuzaki H Toriyama S Toriyama S Kawano H Ohsaka S Scoring system for the preoperative evaluation of metastatic spine tumor prognosis Spine 1990 15 1110 1113 10.1097/00007632-199011010-00005 1702559

[B16] van der Linden YM Dijkstra SP Vonk EJ Marijnen CA Leer JW Dutch Bone Metastasis Study Group Prediction of survival in patients with metastases in the spinal column: results based on a randomized trial of radiotherapy Cancer 2005 103 320 328 10.1002/cncr.20756 15593360

[B17] Tomita K Kawahara N Kobayashi T Yoshida A Murakami H Akamaru T Surgical strategy for spinal metastases Spine 2001 26 298 306 10.1097/00007632-200102010-00016 11224867

[B18] Scheffé H The Analysis of Variance 1959 New York: Wiley

